# Calibrating Data Mismatches in Deep Learning-Based Quantitative Ultrasound Using *Setting Transfer Functions*

**DOI:** 10.1109/TUFFC.2023.3263119

**Published:** 2023-05-25

**Authors:** Ufuk Soylu, Michael L. Oelze

**Affiliations:** Department of Electrical and Computer Engineering and the Beckman Institute, University of Illinois at Urbana-Champaign, Urbana, IL 61801 USA; Department of Electrical and Computer Engineering, the Beckman Institute, and the Carle Illinois College of Medicine, University of Illinois at Urbana-Champaign, Urbana, IL 61801 USA

**Keywords:** Data mismatch, deep learning (DL), tissue classification, transfer function, ultrasound imaging

## Abstract

Deep learning (DL) can fail when there are data mismatches between training and testing data distributions. Due to its operator-dependent nature, acquisition-related data mismatches, caused by different scanner settings, can occur in ultrasound imaging. As a result, it is crucial to mitigate the effects of these mismatches to enable wider clinical adoption of DL-powered ultrasound imaging and tissue characterization. To address this challenge, we propose an inexpensive and generalizable method that involves collecting a large training set at a single setting and a small calibration set at each scanner setting. Then, the calibration set will be used to calibrate data mismatches by using a signals and systems perspective. We tested the proposed solution to classify two phantoms using an L9–4 array connected to a SonixOne scanner. To investigate generalizability of the proposed solution, we calibrated three types of data mismatches: pulse frequency mismatch, focus mismatch, and output power mismatch. Two well-known convolutional neural networks (CNNs), i.e., ResNet-50 and DenseNet-201, were trained using the ultrasound radio frequency (RF) data. To calibrate the setting mismatches, we calculated the *setting transfer functions*. The CNNs trained without calibration resulted in mean classification accuracies of around 52%, 84%, and 85% for pulse frequency, focus, and output power mismatches, respectively. By using the *setting transfer functions*, which allowed a matching of the training and testing domains, we obtained the mean accuracies of 96%, 96%, and 98%, respectively. Therefore, the incorporation of the *setting transfer functions* between scanner settings can provide an economical means of generalizing a DL model for specific classification tasks where scanner settings are not fixed by the operator.

## Graphical Abstract

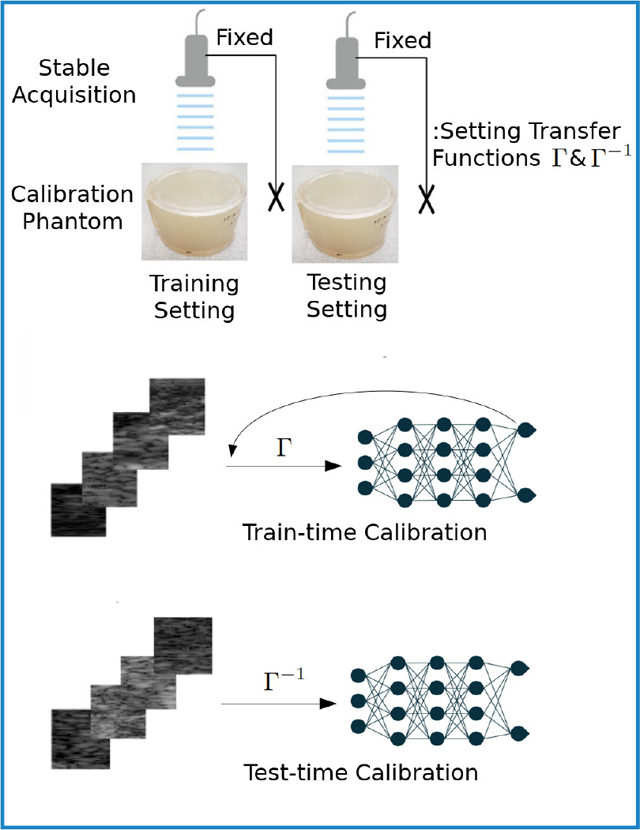

## I. Introduction

Deep learning (DL)-powered biomedical ultrasound imaging is becoming more advanced and coming closer to routine clinical applications in recent years due to a rapid increase in computational power and greater availability of large datasets [1]. DL is the process of learning a hierarchy of parameterized nonlinear transformations to perform a desired function, and therefore, DL extracts a hierarchy of features from raw input images automatically rather than extracting features manually. Among DL algorithms, convolutional neural networks (CNNs) are the most popular structure for ultrasound biomedical imaging, because they are data-efficient learners for image analysis tasks because of their translational invariance [2].

One application of DL in quantitative ultrasound (QUS) is the classification of tissue state from raw radio frequency (RF) ultrasound backscatter. Traditional QUS approaches based on spectral features, i.e., the backscatter coefficient or power spectrum, provide model-based parameters that supposedly correlate to an underlying tissue structure. These parameters are then used to classify tissue state. Popular models, such as fitting a straight line to the backscatter coefficient or using a Gaussian model, do not actually describe real physical tissue structures; i.e., the models are incorrect. The ultimate goal is to classify tissue state, which could include distinguishing between benign and malignant masses. On the other hand, DL-based QUS can directly classify the tissue state without needing a model to parameterize the signal. Therefore, QUS has recently evolved from model-based approaches [3], [4] to model-free, DL-based techniques [5], [6], [7], [8].

In an initial work, a CNN was used to classify liver steatosis in rabbits based on the RF backscattered data [6]. The advantage of the approach was that the CNN learned the tissue signal and separated it from the system signal. Therefore, the CNN approach did not require a separate calibration spectrum for analysis and did not need to fit the data to a preconceived model; i.e., the CNN learned the model. This CNN approach was then compared with a traditional QUS approach in the characterization of fatty liver with better performance attributed to the CNN [7]. Similar approaches were later used to quantify liver disease in human patients and to characterize breast masses for cancer detection [9], [10], [11]. Similar to these previous works, this study utilizes a DL approach to classify tissue state, or specifically in this case phantom identity, based on the RF backscattered data.

Even though DL is promising for classification of tissues based on their RF backscattered data, there are two main road-blocks to wider clinical adoption as stated by Castro et al. [12]. One road block to deploying DL-powered algorithms to real clinical settings is data scarcity. Specifically, there is scarcity of labeled data, in great part due to high costs of conducting laboratory experiments or acquiring expert annotations. The other main road block is data mismatch. Data mismatch happens due to mismatches between development and deployment environments and tends to limit generalizability of DL-based algorithms. Overall, in the generic case, when there is not enough labeled data or when no assumptions can be made about the mismatches between development and deployment settings, any learning-based algorithm would be ineffective. Therefore, to turn DL-powered biomedical ultrasound imaging into reality, there is a constant need for developing DL algorithms, which are data-efficient and more robust against data mismatches in ultrasound images.

The clinical environment is too complicated to be fully realized in a development setting where operators, such as sonographers, are adjusting settings to obtain the best perceived image quality. Therefore, there will be inevitable data mismatches if the operator is given the freedom to select settings that provide perceived optimal image quality, and these settings do not match scanner settings associated with the training data. Specifically, we consider acquisition-related data mismatches, i.e., data mismatches caused by variations in scanner settings, such as the number of foci and their locations or pulse frequency, and develop a method by looking at the problem from a signals and systems perspective. Such data mismatches are pervasive in biomedical ultrasound imaging due to its operator-dependent and patient-dependent nature, and mitigating their effects is essential for wider clinical adoption of DL-based methods for tasks, such as tissue classification.

Transfer learning (TL) is a technique that aims to address the issue of data mismatch by first training a learning model and then fine-tuning it [13], [14]. TL is potentially more costly than the proposed calibration method, because it requires diverse sample data in the testing domain to fine-tune a model. In the proposed method, a single frame from a single calibration source from the testing domain is sufficient. The problem of data mismatch has also become more prominent in recent literature on DL-based QUS [15], [16], [17], [18], [19]. Therani et al. [16] utilized reference phantoms, which have known scatter number density to mitigate system dependency in the problem of classifying scatterer number density through adaptive batch normalization. In contrast, the proposed calibration method employs a single reference phantom that is not dependent on the type of classes. In another interesting work, Sharifzadeh et al. [19] proposed replacing the magnitude of the low-frequency spectrum inspired by Fourier domain adaption (FDA) in the field of computer vision. Unlike that work, the method proposed here is capable of utilizing the entire frequency spectrum by requiring only a single frame from the testing domain. In addition, Tierney et al. [18] used cycle-consistent generative adversarial networks to eliminate data mismatches in ultrasound images. It should be noted, however, that generative models require a larger amount of diverse data in the testing domain, making them more resource-intensive in comparison with the method proposed in this article.

In this work, we propose a method to mitigate the effects of data mismatch in biomedical ultrasound imaging with the specific task of characterizing tissues based on raw RF data derived from ultrasonic backscatter. The proposed method is an inexpensive way of mitigating generalizability issues caused by acquisition-related data mismatches in biomedical ultrasound imaging. In our experiments, DL approaches were trained to classify raw RF data to identify distinct tissue-mimicking phantoms. Traditional QUS approaches can account for system- and operator-dependent changes to the settings through calibration of the system at each setting, which requires the use of models and reference phantoms with known characteristics [20], [21]. However, these models are not accurate for real tissue. On the other hand, DL-based QUS techniques typically use a single scanner setting. This work shows that a reference phantom can be used to calibrate the setting mismatch in DL-based techniques similar to spectral-based QUS approaches. Overall, we use DL algorithms with the backscattered RF ultrasound signal to utilize the phase- and frequency-dependent information for the tissue classification problem without the constraint that the settings be the same for each scan. The theoretical bases for this approach are discussed in II. Further details of our experiments can be found in Sections III and IV. We provide discussions and conclusions in Sections V and VI, respectively.

## II. Theory

Any form of learning algorithm would be ineffective if there are data mismatches between training and testing data, and no assumptions can be made to calibrate the mismatches. Ideally, we need to collect a large and diverse training dataset at each imaging setting to completely eliminate data mismatches caused by scanner parameters. However, acquiring such a training dataset can be extremely expensive. Another approach could be training on a subset of imaging settings, which makes the data generation and gathering process less expensive. However, there will still be generalization issues for the settings that are not included in the training set. The question we address here is “How can we mitigate acquisition-related data mismatches in an inexpensive and generalizable way for QUS?”

To accomplish this, we consider a systems response approach. Biomedical ultrasound imaging can be viewed as a system, which encodes all the information related to an imaging system working on a tissue signal, which encodes all the information related to an imaging substrate. Subsequently, an image obtained by an ultrasound imaging system can be decomposed into two parts: a system response and a tissue signal. Then, the problem of acquisition-related data mismatch can be posed as matching system responses. When training inputs and testing inputs are from different distributions due to different scanner settings, such as a different excitation pulse frequency, there are two different system responses in the imaging process, one corresponding to training and the other one corresponding to deployment or testing. Potentially, a function can be defined that allows a system to transfer from one environment to the other environment.

More precisely, under a single scattering approximation and when at least one aperture diameter away from the transducer surface, the backscattered frequency spectrum from a medium can be represented as follows [22]:

(1) $$W\left(f,\;x\right)=T\left(f,\;x\right)A\left(f,\;x\right)D\left(f,\;x\right)H\left(f\right)R\left(f,\;x\right)$$

where f represents the frequency, x represents the axial direction, T(f,x) incorporates the transmission losses between tissues, A(f,x) is the frequency-dependent attenuation, D(f,x) represents the diffraction effects of the transducer, H(f) is the impulse response of the transducer system and incorporates the electromechanical response, and R(f,x) is the scattering function describing the underlying tissue microstructure. Therefore, an ultrasound image can be naively decomposed as follows:

(2) $$W\left(f,x\right)=S_{\phi }\left(f,x\right)P\left(f,x\right)$$

where $S_{\phi }(f,x)=D(f,x)H(f,x)$ is the system response, which incorporates all the information related to ultrasound imaging system, and P(f,x)=T(f,x)A(f,x)R(f,x), which is the tissue or sample signal that incorporates all the information related to imaging substrate (i.e., attenuation, transmission losses, and scattering function). The subscript ϕ in $S_{\phi }(f,x)$ represents the scanner setting, exclusively $\phi_{\text{train}}$ stands for training environment and $\phi_{\text{test}}$ stands for testing environment, for later use. To calibrate the acquisition-related data mismatches between two system settings, we setup the scenario where the tissue signal, P(f,x), does not change between testing and training, such that

(3) $$\frac{W_{test}(f,\;x)}{W_{\text{train}}(f,\;x)}=\frac{S_{\phi_{test}}(f,\;x)}{S_{\phi_{train}}(f,\;x)}$$

(4) $$=\Gamma \left(f,x\right)$$

where Γ(f,x) represents the *“setting transfer function”* between training and testing system parameters. This can be done in a practical way by selecting a tissue mimicking phantom with uniform scattering properties and fixing the transducer to scan and record the signal from a single location in the phantom, while the settings are changed from training to testing. To calibrate in training time, Γ(f,x) is sufficient

(5) $$W_{\text{test}}\left(f,x\right)=\Gamma \left(f,x\right)W_{\text{train}}\left(f,x\right)$$

and hence, it is a convolution operation in time direction, t

(6) $$w_{\text{test}}\left(t,x\right)=\gamma_{train}\left(t,x\right)\;{*}_{t}\;w_{train}\left(t,x\right)$$

where $\gamma_{train}$ represents the *“setting transfer filler”* for training time. For the train-time calibration, a DL model is trained at testing data settings, and therefore, testing data can be input into the DL model directly. In order to achieve this, the training data are converted to the testing data via the setting transfer function Γ(f,x) in training time, and a new model is developed by the network, which allows the testing data settings to match with training settings used to create the model.

Similarly, $\Gamma^{-1}(f,x)$ is sufficient for calibrating in testing time

(7) $$W_{train}\left(f,x\right)=\Gamma^{-1}\left(f,x\right)W_{test}\left(f,x\right)$$

which results in the following filtering operation:

(8) $$w_{\text{train}}\left(t,x\right)=\gamma_{\text{test}}\left(t,x\right)\;{*}_{t}\;w_{\text{test}}\left(t,x\right)$$

where $\gamma_{\text{test}}$ represents the *“setting transfer filter”* for testing time. For the test-time calibration, a DL model is trained at the training data settings, and therefore, testing data cannot be input into the DL model directly. The testing data need to be converted to training data via the setting transfer function $\Gamma^{-1}(f,x)$. Then, the model developed with the original training data and its associated settings are used with the converted testing data.

In this work, we propose to use a signals and systems perspective to *calibrate* training of testing data. Here is the proposed method step by step at a high level.

1. Gather a *large* amount of training data at a single setting.
2. Gather a *small* amount of calibration data at each scanner setting.
3. Calculate $W_{\text{test}}$ or $W_{\text{train}}$ by using the calibration set to calculate the setting transfer function Γ and $\Gamma^{-1}$.
4. Construct linear phase filters $\gamma_{train}$ or $\gamma_{\text{test}}$ by using the magnitude responses of Γ and $\Gamma^{-1}$.
5. When a different setting than training setting is being used in the scanning process, calibrate the mismatch by using filters $\gamma_{\text{train}}$ or $\gamma_{\text{test}}$ either in the training time or in the testing time in the DL network, respectively.

## III. Methods

### A. Phantoms

**Fig. 1.**
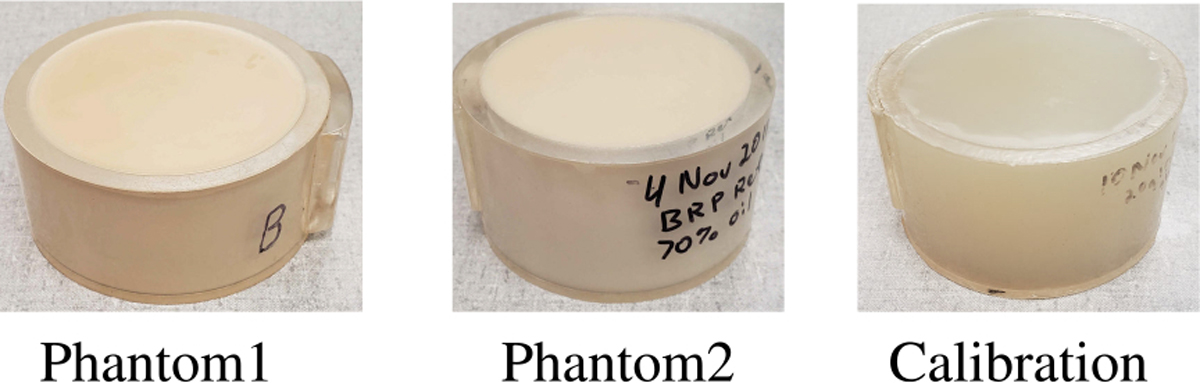
Photographs of the classification and calibration phantoms.

Two different tissue-mimicking phantoms were classified in the experiments, which we designated as Phantom 1 and Phantom2, and another phantom was used as the calibration phantom. They are cylindrically shaped, as shown in Fig. 1.

Phantom 1, which mimics soft tissue, has been described by Wear et al. [23]. Their materials were produced based on the method of Madsen et al. [24], and they are macroscopically uniform. The only nonuniformity results from the random positioning of microscopic glass bead scatterers. Phantom 1 had a measured attenuation coefficient slope of approximately $0.7\;dB\times {cm}^{-1}\times {MHz}^{-1}$, respectively. The component materials and their relative amounts by weight for Phantom 1 are agarose (2.34%), n-propanol (2.92%), 75–90-μm-diameter glass beads (1.87%), bovine milk concentrated three times by reverse osmosis (47.9%), liquid Germall Plus preservative (International Specialty Products, Wayne, NJ, USA) (1.87%), and 18-MΩ-cm deionized water (43.1%).

Phantom2 has been described by Nam et al. [20] as a reference phantom. Its measured attenuation coefficients at frequencies from 2 to 10MHz were fit to a power law function of frequency, $\alpha (f)=0.256{\;f}^{1.366}$, where f is the frequency in terms of MHz and α(f) is in terms of dB/cm. The phantom was made with 6.4 g of 5-43–μm-diameter glass beads uniformly distributed spatially at random in a gel background. The background material was a gelatin emulsion containing 70% safflower oil [25].

The calibration phantom was a low attenuation phantom, which was constructed as described by Anderson et al. [26]. It had a weakly scattering 2% agar background with 150–180-*μ*m glass beads, which had a slightly broader distribution of scatterer sizes (160 ± 60 *μ*m). The glass bead concentration was 20 g/L, and the beads were randomly distributed spatially within the phantom.

### B. Ultrasound Imaging Device and Imaging Settings

Ultrasound gel was placed on the surfaces of the phantoms, and then, the phantoms were scanned with an L9–4/38 transducer using a SonixOne system (Analogical Corporation, Boston, MA, USA) providing an analysis bandwidth of 2–7.5 MHz and focusing with an F-number of 3 for transmit and receive. Ultrasound frames of post-beamformed RF data sampled at 40 MHz were acquired from each of the phantoms and saved for offline processing. The ultrasound post-beamformed RF data were directly used in the training, test, and calibration processes. The imaging array had a center frequency measured at 5.5 MHz and was operated with a fixed elevational focus of 1.9 cm. Scanner parameters that were adjusted for each experiment can be found in Table I. Changes in the focus occurred on transmit. We acquired data from the phantoms via two scanning procedures. In the first, we recorded a video of 1007 ultrasound frames by free-hand motion. Free-hand acquisition provided us a large dataset of independent frames for each phantom to be used in the training and testing. In the second procedure, we stabilized the transducer using a bar clamp holder and then recorded ten identical frames at both the training and testing settings from the exact same location in the phantom, which provided us with the calibration data to be used in calculating the setting transfer functions.

### C. Dataset

The total size of an ultrasound image frame from the phantoms was 2080 × 256 pixels. There were 2080 samples along the axial direction that corresponded to a 4-cm imaging depth. Even though the L9–4/38 transducer has 128 channels, the SonixOne system interpolates to 256 channels that correspond to 256 lateral samples. The data used in the DL network were the raw backscattered RF data. The dataset of ultrasound frames is also publicly available at https://figshare.com/s/7ae94a537a56e5db3525.

After acquiring ultrasound frames by either stable acquisition or free-hand acquisition, we extracted square data patches from the image frames whose sizes were 200 × 26 samples that correspond to square image patches whose sizes were 4 × 4 mm in physical dimensions, to be used in training, validation, and testing sets. The motivation behind patch extraction was described in our previous work through a clinical scenario [8]. For instance, ultrasound imaging can be used to examine and characterize tumors, whether benign or malignant. When using QUS approaches for tumor characterization, a region of interest (ROI) is selected inside the tumor to examine the signals from the tumor. Therefore, we performed the patch extraction in this work to examine the proposed method in the context of DL-based QUS.

**Fig. 2.**
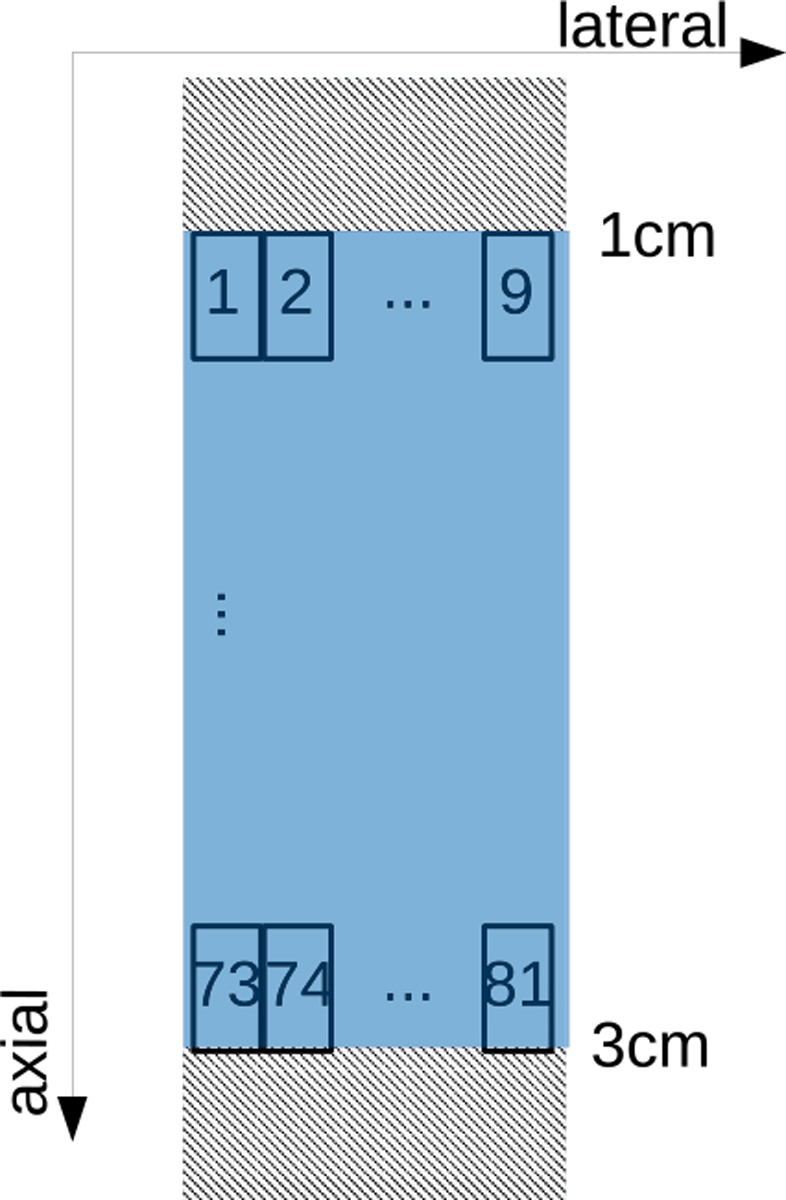
Patch extraction: local patches, whose sizes were 200 × 26 samples, were extracted to be input into a CNN. The first 540 samples were not used. Each frame resulted in 81 extracted patches due to the nine axial and nine lateral lines used for patch extraction.

From one ultrasound image, we could extract 81 (9 lateral × 9 axial) image patches, as depicted in Fig. 2. While extracting image patches, we did not use the first 540 pixels in the ultrasound image. Axially, we obtained the next line of individual patches by translating the start of the next patch by 100 pixels along the axial depth. Laterally, we obtained the next line of individual patches by translating the start of the next patch by 26 pixels along the axial depth. Overall, in patch extraction, there were nine axial lines and nine lateral lines to extract individual patches that led to extracting 81 image patches per ultrasound image. As a consequence, the training set consisted of image patches extracted from ultrasound frames acquired at scanner settings for the training. On the other hand, ultrasound frames acquired for the test data were split into two sets: one was for the validation set, and the other was for the test set.

In training, we extracted 81000 patches, which is equal to 1000 frames × 81 patches per frame. The 1000 frames were randomly selected out of 2014 total ultrasound frames at the scanner setting for the training. Similarly, after acquiring 2014 frames at scanner settings for the testing, as the validation and test sets, we randomly selected 750 ultrasound frames out of 2014 ultrasound frames, which resulted in 60750 patches for validation and testing. We repeated the random selection of training and testing patches ten times for each experiment.

### D. Network Structure

In this work, we used two well-known CNN architectures, i.e., ResNet-50 [27] and DenseNet-201 [28]. CNNs have several advantages among other DL structures for the tasks related to 2-D images. CNNs are similar to the human visual system, which makes them effective at learning and extracting abstractions of 2-D images [29]. The CNN architectures were slightly modified and can be found in Tables II and III.

### E. Training

The DL training was done on a machine having a TITAN RTX and on two machines each having an RTX A5000. Each experiment was conducted separately on one of the three available GPUs. All implementations were done with the PyTorch library [30].

As a data preprocessing step, we applied z-score normalization at the patch level; i.e., the mean intensity value of patches was subtracted from each patch, and then, each pixel in a patch was divided by the standard deviation of the intensity of the patches. Then, the models were trained by using cross-entropy loss with uniform class weights. Horizontal flip with 0.5 probability was implemented as a default data augmentation step in the training process. The batch number was chosen as 128 for all experiments. We used the Adam algorithm [31] as the optimizer in all experiments. The learning rates and the epoch numbers were determined to achieve “asymptotic test accuracy” by using the validation set.

After adjusting all the training parameters, we repeated training and testing for each experiment ten times starting from random selection of ultrasound frames and dividing them into patches. Then, we calculated the mean classification accuracies and standard deviations in the test set. Also, we calculated mean area under the receiver operator characteristic curve (AUC) and standard deviations. The metrics are calculated patch-wise and reported in Section IV for each experiment.

### F. Calibration

We first obtained $W_{\text{train}}(f,x)$ and $W_{\text{test}}(f,x)$, defined in (3) by using the calibration set obtained at testing and training settings. We gathered the calibration set by stabilizing the transducer array on top of a phantom and acquiring scans from the exact same view for each setting. For the training and testing settings, we acquired ten identical frames from the calibration phantom to be used in averaging and reducing any systematic noise. Because of the stable acquisition setup, i.e., using the same tissue signal P(f,x) in the calibration set, by taking ratios of $W_{\text{train}}(f,x)$ and $W_{\text{test}}(f,x)$, we obtained setting transfer functions Γ(f,x) and $\Gamma^{-1}(f,x)$. The ratios can be used either in training time by applying Γ(f,x), as shown in (5), which we call “*train-time calibration*”, or in test time by applying $\Gamma^{-1}(f,x)$, as shown in (7), which we call “*test-time calibration*”.

In practice, we implemented the setting transfer functions in a manner inspired by the Wiener filter [32]

(9) $$\Gamma_{\text{Wiener}}(f,\;x)=\frac{\frac{1}{\left|\Gamma (f,x)\right|}}{\frac{1}{|\Gamma (f,x){|}^{2}}\;+\;\frac{1}{SNR}}$$

(10) $$\Gamma_{\text{Wiener}}^{-1}(f,\;x)=\frac{\left|\Gamma (f,\;x)\right|}{|\Gamma (f,\;x){|}^{2}\;+\;\frac{1}{SNR}}$$

where SNR was estimated through the power spectra of $W_{\text{train}}$ and $W_{\text{test}}$. First, we determined a noise floor level by looking at the lowest values of the power spectra outside of the transducer bandwidth, i.e., around 20MHz. Then, at each frequency bin, we calculated the tissue signal level by subtracting the noise floor. Subsequently, we obtained SNR values by taking the ratios of the tissue signal and the noise floor at each frequency bin for both the power spectra of $W_{\text{train}}$ and $W_{\text{test}}$. Finally, we took the minimum SNR value between $W_{\text{train}}$ and $W_{\text{test}}$ used that value in the filter. In this filter design, at frequency bins when the signal level of the setting transfer function was small compared with the noise, the filter acted like a denoising filter. When the signal level of the setting transfer function was high compared with noise, the filter was equivalent to the original Γ. This provides a robust way to use the complete bandwidth of setting transfer functions.

In the patch extraction, patches originated from nine different depth or axial lines as described in Section III-C. Therefore, the power spectra $W_{\text{train}}$ and $W_{\text{test}}$, and, hence, the setting transfer functions $\Gamma_{\text{Wiener}}$ and $\Gamma_{\text{Wiener}}^{-1}$, were obtained in a depth aware manner; i.e., transfer functions were calculated for each axial line (i.e., each depth), resulting in nine transfer functions per setting mismatch. After calculating $\Gamma_{\text{Wiener}}(f,x)$ and $\Gamma_{\text{Wiener}}^{-1}(f,x)$, $\gamma_{\text{train}\;}$ from (6) and $\gamma_{\text{test}\;}$ from (8) were constructed as finite impulse response (FIR) filters with linear phase from the given frequencies and corresponding gains. The number of taps in the FIR filter was searched in the hyperparameter optimization and selected as 51.

FIR filters were constructed by using scipy.signal.firwin2 function from Python. The implementations of convolution operations in (6) and (8) were done via torch.nn.functional.conv ld whose parameter padding was selected as “same,” which pads the input, so the output has the same shape as the input. The implementation code can be found at https://github.com/usoylu2/calibration.

*Train-time calibration* and *test-time calibration* are explained in Algorithms 1 and 2, respectively. From Figs, 3–5, we plotted power spectra for different training–testing setting pairs corresponding to Table II along with the transfer functions Γ(f,x) and $\Gamma_{\text{Wiener}}(f,x)$. These graphs are obtained from data acquired at a fixed axial location, which is around 2 cm. The left subfigures show power spectra $W_{\text{train}}$ and $W_{\text{test.}}$ Furthermore, the right subfigures depict Γ(f,x), $\Gamma_{\text{Wiener}}(f,x)$, and Fourier Transform of the linear phase filter $\gamma_{\text{train}}$.

### G. Transfer Learning

In this work, we used TL as the baseline method. We fine-tuned CNNs, which had been trained using a large amount of data acquired from the samples at the training setting, by using a smaller amount of data acquired from the samples using the testing setting. Specifically, we compared the proposed method with TL for three different dataset sizes after training CNNs with the complete training set, which consisted of 1000 frames. For the first set, we fine-tuned the network using two diverse frames (162 patches) in training, two diverse frames in validation, and two diverse frames in testing. For the second, we used ten diverse frames (810 patches) in training, ten diverse frames in validation, and ten diverse frames in testing. For the third, we used 20 diverse frames (1620 patches) in training, 20 diverse frames in validation, and 20 diverse frames in testing. The major limitation for adopting the TL method, in comparison to the proposed method, is that it requires acquisition of a set of diverse frames from the actual samples at the testing settings to be transferred to the model that was developed with data acquired using the training settings.

## IV. Results

### A. Pulse Frequency Mismatch

First, we investigated whether transfer functions could mitigate the effects of a frequency mismatch. We acquired the training data at a 9-MHz pulse frequency setting and the testing data at a 5-MHz pulse frequency setting.

In Table IV, *“train-time calibration”* and *“test-time calibration”* for the pulse frequency mismatch are compared with a benchmark experiment named “benchmark” in which there was no mismatch, i.e., the same pulse frequency was used for the training and testing data, to an experiment named “no calibration” in which we did not use any calibration and to the baseline method TL. For *train-time calibration*, there were two experiment types: “train-time calibration (50%)” in which 50% of the training data are calibrated, and the remaining 50% of the training data are uncalibrated, and “train-time calibration (100%)” in which 100% of the training data are calibrated. For the baseline method TL, there were three experiment types: “TL with two frames” in which the training set consists of two diverse frames, “TL with ten frames” in which the training set consists of ten diverse frames, and “TL with 20 frames” in which the training set consists of 20 diverse frames. For *train-time calibration* and *test-time calibration*, learning rates were 5*e* – 5 and 1*e* – 4, and epoch numbers were 20 and 30. For TL experiments, learning rate was 2*e* – 6, and epoch number was 20. For “no calibration,” learning rate was 1*e* – 6, and epoch number was 20. For “benchmark,” learning rates were 5*e* – 5 and 1*e* – 5, and epoch number was 25.

### B. Focus Mismatch

In this section, we investigated whether transfer functions could mitigate effects of a focus mismatch. We acquired the training data focused at 2 cm and the test data with dual foci at 1 and 3 cm.

Similar to the case with the pulse frequency mismatch, in Table V, *“train-time calibration”* and *“test-time calibration”* for the focus mismatch are compared with “benchmark,” “no calibration,” and TL experiments. For *train-time calibration* and *test-time calibration*, learning rates were 5*e* – 5, 5*e* – 6, and 1*e –* 5, and epoch numbers were 20 and 25. For TL experiments, learning rate was 2*e* – 6, and epoch number was 20. For “no calibration,” learning rate was 5*e* – 5, and epoch number was 20. The “benchmark” was the same as Section IV-A.

### C. Output Power Mismatch

Finally, we investigated if we could mitigate effects of a data mismatch of output power by using transfer functions. We acquired the training data by using 0-dB output power, which represents the maximum output power of the imaging system, and the test data by using −6-dB output power, which represents the output power level that is 6 dB below the maximum.

Similar to the case with the pulse frequency mismatch, in Table VI, *“train-time calibration”* and *“test-time calibration”* for the output power mismatch are compared with “benchmark,” “no calibration,” and TL experiments. For *train-time calibration* and *test-time calibration*, learning rates were 5*e* – 5 and 1*e* – 5, and epoch numbers were 20 and 25. For TL experiments, learning rate was 2*e* – 6, and epoch number was 20. For “no calibration,” learning rate was 5*e* – 5, and epoch number was 20. The “benchmark” was the same as Sections IV-A and IV-B.

## V. Discussion

**Fig. 3.**
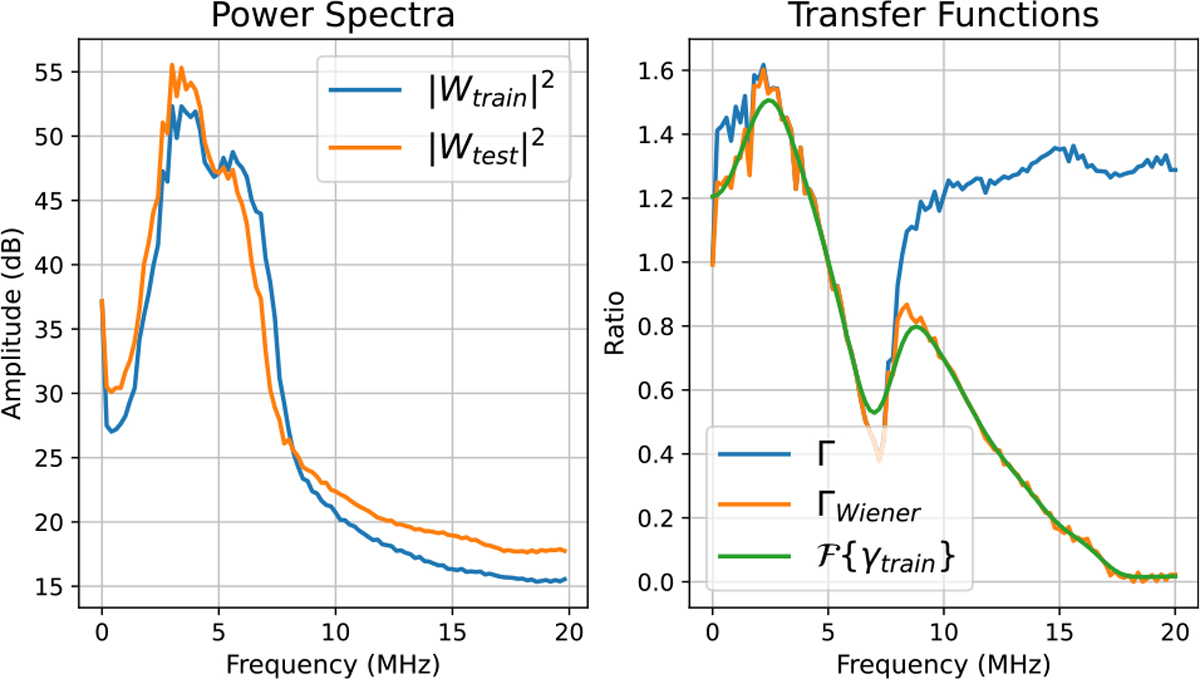
Example calibration plots for the pulse frequency mismatches.

**Fig. 4.**
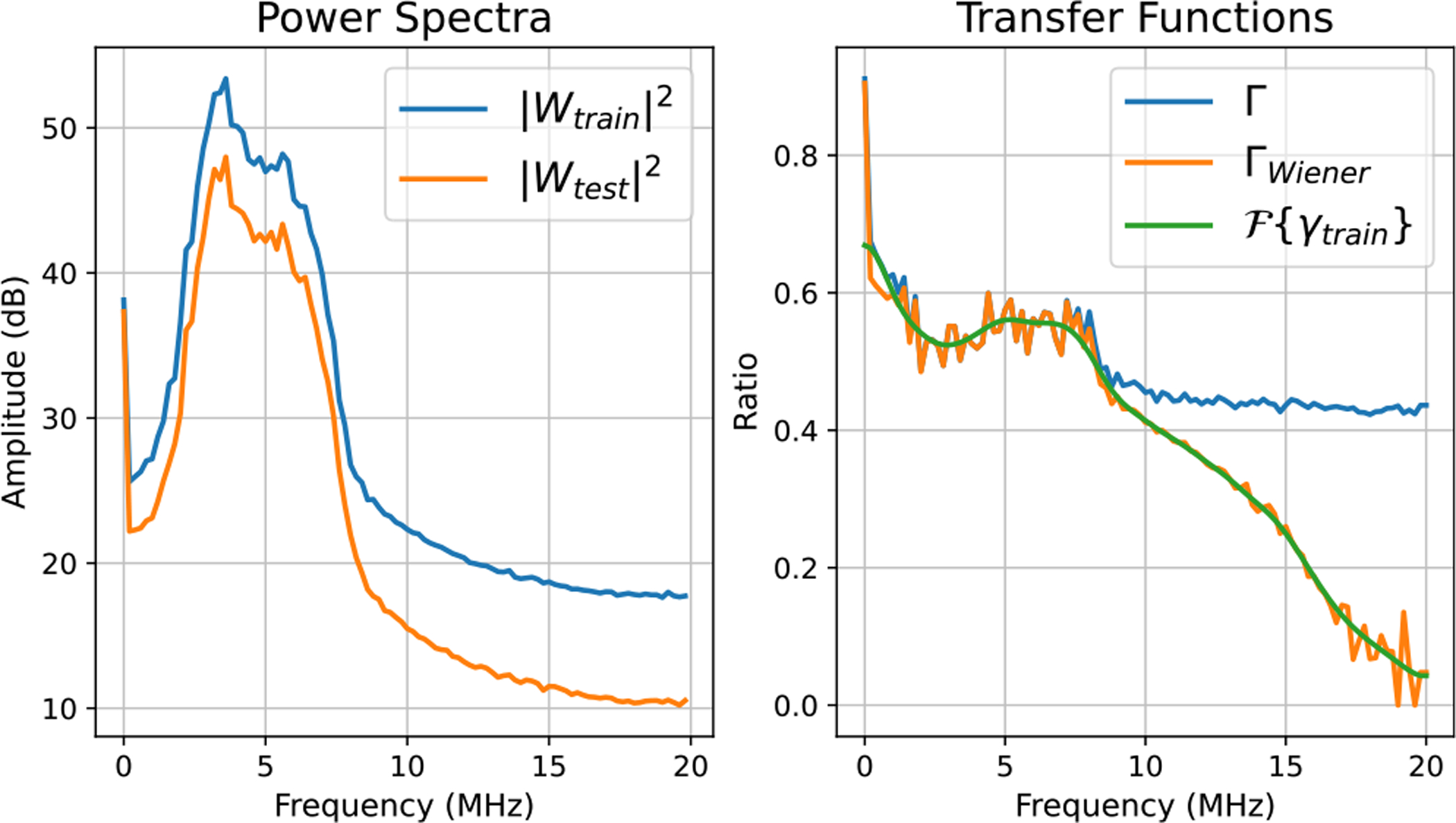
Example calibration plots for focal location mismatches.

**Fig. 5.**
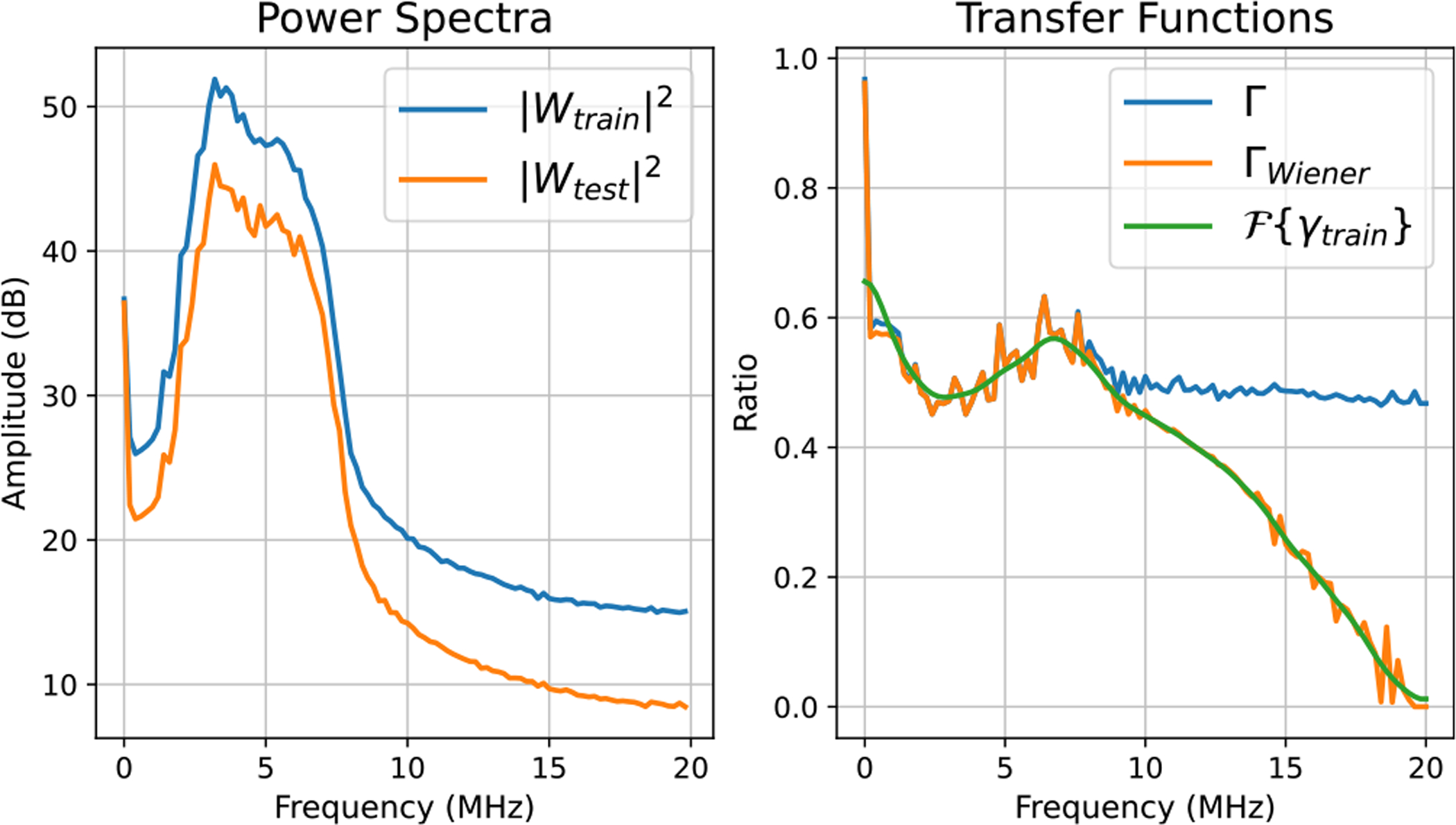
Example calibration plots for the output power mismatches.

From Figs. 3–5, the effect of the data mismatches on the averaged power spectrum is visualized. In Fig. 3, the averaged power spectrum of the data from the training setting was shifted to higher frequencies in comparison with the averaged power spectrum of the data from the testing setting. That is expected, because we used a 9-MHz pulse frequency in the training setting and a 5-MHz pulse frequency in the test setting. However, the actual shift in the spectrum was relatively narrower than 4 MHz. Overall, while the averaged power spectrum of the data from the testing setting was shifted to lower frequencies, the averaged power spectrum of the data from the training setting was shifted to a higher frequency. That led to the setting transfer functions Γ and $\Gamma_{\text{Wiener}}$ to be greater than unity below 5 MHz and less than unity above 5 MHz. $\Gamma_{\text{Wiener}}$ matched Γ well around the analysis bandwidth due to high SNR and rapidly goes to zero above 10 MHz.

In Fig. 4, the averaged power spectrum of the data from training setting had higher amplitude than the averaged power spectrum of the data from testing setting, because those plots were obtained around 2 cm axially, which corresponds to the focal region of the training setting. As a result, the setting transfer functions Γ and $\Gamma_{\text{Wiener}}$ were approximately constant at 0.6 around the analysis bandwidth. Similarly, in Fig. 5, the averaged power spectrum of the data from the training setting had higher amplitude than the averaged power spectrum of the data from the testing setting. That is due to using 6-dB higher output power in the data acquisition, which led to the setting transfer functions Γ and $\Gamma_{\text{Wiener}}$ to be relatively constant at 0.5 around the analysis bandwidth. Similar to Fig. 3, in Figs. 4 and 5, $\Gamma_{\text{Wiener}}$ approached zero above 10 MHz due to low SNR.

In Table IV, we observed that the proposed method mitigated the effects of the given frequency mismatch. ResNet and DenseNet trained without any calibration resulted in the mean classification accuracies of 52.35% and 52.33%, respectively, which are equivalent to random guess classifiers. When the proposed method was applied, we obtained mean classification accuracies of 96.77% and 95.45%, respectively, which are substantially closer to the benchmark performance. In terms of AUC, ResNet and DenseNet without any calibration resulted in the mean AUC of 0.927 and 0.938, respectively. When the proposed method was applied, mean AUCs were 0.996 and 0.994, respectively. In comparison with the baseline method, the proposed method was more data-efficient, as it used a single frame to calibrate the mismatch, while TL needed ten diverse training frames and ten diverse validation frames to catch up with the proposed method in terms of accuracy, and TL needed 20 diverse training frames and 20 diverse validation frames to achieve similar performance as the proposed method in terms of AUC. In addition, *train-time calibration* performed better than *test-time calibration* for the given mismatch in terms of both accuracy and AUC. Furthermore, when we applied *train-time calibration* for all the training data (calibration 100%) performed better than applying *train-time calibration* for half of the training data (calibration 50%). This indicates that calibrated training data provided all the potential performance increase. Using mismatched (uncalibrated) training data did not provide any additional performance increases for the given experiment.

In Table V, we observed that the proposed method mitigated the effects of the given focus mismatch. ResNet and DenseNet trained without any calibration resulted in the mean classification accuracies of 83.44% and 85.52%, respectively. When the proposed method was applied, we obtained the mean classification accuracies of 96.67% and 96.34%, respectively. In terms of AUC, ResNet and DenseNet without any calibration resulted in the mean AUC of 0.929 and 0.939, respectively. When the proposed method was applied, mean AUCs were 0.997 and 0.996, respectively. Similar to the frequency mismatch, in comparison with the baseline method, the proposed method is more data-efficient, as it used a single frame to calibrate the mismatch. Unlike the frequency mismatch, *test-time calibration* performed better than *train time calibration* and using mismatched training data provided additional performance increases for the given experiment.

In Table VI, we observed that the proposed method mitigated the effect of the given output power mismatch. ResNet and DenseNet trained without any calibration resulted in the mean classification accuracies of 86.98% and 84.41%, respectively. When the proposed method was applied, we obtained the mean classification accuracies of 98.99% and 98.39%, respectively. In terms of AUC, ResNet and DenseNet without any calibration resulted in the mean AUC of 0.957 and 0.923, respectively. When the proposed method was applied, mean AUCs were 0.999 and 0.999, respectively. Similar to the results from the previous mismatches, in comparison with the baseline method, the proposed method is more dataefficient. Similar to the results from the focal mismatch, *test-time calibration* performed slightly better than or close to *train-time calibration*, and using mismatched training data provided additional performance increases for the given experiment.

Regarding *test-time calibration* versus *train-time calibration*, we observed that they were relatively comparable in terms of AUC. However, in terms of accuracy, *train-time calibration* performed better for the frequency mismatch and *test-time calibration* performed better for the focal and the output power mismatches. One advantage of *test-time calibration* over *train-time calibration* is its simplicity. The *train-time calibration* requires the training data to be converted, added to the data, and the model retrained. With the *test-time calibration*, there is no need for any retraining or fine-tuning of the network when a new test setting is being used. Therefore, the *test-time calibration* approach is more suitable for real-time clinical applications. On the other hand, *train-time calibration* has some algorithmic advantages, such as choosing hyperparameters being more convenient, because the training error is directly related to validation error.

In the three types of mismatches, we consistently observed a small decrease in AUC, while the classification accuracy dropped significantly. For example, in the frequency mismatch case, the classification accuracy dropped from 99% to 52%, while the AUC dropped from 0.99 to 0.93. These observations indicate that the separability between the two classes remains high, even when the optimal threshold changes significantly. Therefore, this suggests that the DL network could be calibrated through AUC analysis to identify the new optimal threshold. However, this process requires data to be acquired from actual samples under testing conditions for training similar to TL. Overall, the proposed method increased accuracy significantly and improved AUC, which verifies its calibration capabilities without the need for data acquired from actual samples under testing conditions.

Among the investigated acquisition-related data mismatches, the frequency mismatch led to the largest drop in accuracy for the no calibration case compared with the focal and output power mismatches. Specifically, the mean classification accuracy dropped from 99% to 52% for the frequency mismatch, while it dropped from 99% to 84% for the focal and output power mismatches. These observations suggest that the frequency mismatch caused the most disturbance to the optimal threshold.

Using uncalibrated data in the *train-time calibration* improved accuracy for the focal and output power mismatch but not for the frequency mismatch. Therefore, depending on the type of applications and data mismatches, using uncalibrated data in training could potentially provide richer training data and better generalizability. The proposed method resulted in similar performance improvements for both ResNet and DenseNet, verifying its validity for various network structures. In addition, the proposed method was more data-efficient than the baseline method, as it only required a single calibration view, while the baseline method required ten diverse frames in the training and ten diverse frames in the validation to perform similarly in terms of accuracy, and required 20 diverse frames in the training and 20 diverse frames in the validation to perform similarly in terms of AUC.

Moreover, the proposed method was more practical than the baseline method in the clinical workflow. As it does not require any diverse calibration views, the calibration set can be acquired through automation by stable acquisition. Another point related to the clinical workflow is the need for actual samples. The proposed method does not require actual samples to calibrate the data mismatches, because the calibration phantom is sample-irrelevant. However, TL requires real sample data to fine-tune the models, and it demands more data, as the number of classes increases.

One important future work would be related to how to select the calibration phantom. When nonlinearities in the system and imaging substrate were negligible, the system response of an ultrasound system and, hence, the setting transfer function, Γ, should be the same irrespective of the imaging substrate. Therefore, we could use any phantom with uniform scattering properties as the calibration phantom. However, due to random spatial variation noise from the subresolution scatterers, there could be some fluctuation in the setting transfer function Γ, as it can be observed from Figs. 3–5. If one could average multiple views, variation in the power spectra would decline. However, it would also increase the complexity of the approach. In the proposed approach, we acquired multiple frames of the same view after stabilizing the transducer for the calibration data. This way, the acquisition of the calibration data could be automated easily for all imaging settings without any human intervention. The need of multiple views in the calibration data would make it more complicated and expensive. In addition, further research is needed to investigate the use of a more diverse probe model and imaging settings. As long as the frequency spectrum of training conditions and testing conditions overlaps, the proposed method is expected to work well. Moreover, the proposed method should be investigated for different DL tasks and QUS tasks to fully realize its utility. While the proposed method primarily addresses calibration for estimating QUS parameters related to the frequency domain, its effectiveness for envelope-based parameters, such as scatterer number density and coherent to diffuse scattering power, has not been tested.

## VI. Conclusion

We demonstrated that the proposed approach for mitigating the effects of data mismatches was effective for tissue classification under various mismatches in training versus testing scanner settings: a pulse frequency mismatch, a focal region mismatch, and an output power mismatch. Therefore, the incorporation of transfer functions between scanner settings can provide an economical means of generalizing a DL model for the specific imaging tasks where scanner settings are not fixed by the operator.

## Figures and Tables

**TABLE I:** Scanner Parameters for the SonixOne System

	Pulse Freq	Focus	Output Power

Training in Sec.IV-A	9 MHz	@2cm	0 dB
Test in Sec.IV-A	5 MHz	@2cm	0 dB

Training in Sec.IV-B	9 MHz	@2cm	0 dB
Test in Sec.IV-B	9 MHz	@1cm and 3cm	0 dB

Training in Sec.IV-C	9 MHz	@2cm	0 dB
Test in Sec.IV-C	9 MHz	@2cm	−6 dB

**TABLE II:** ResNet-50

stage	output	kernels

conv1	100 × 13	7 × 7, 64, stride 2

		3 × 3 max pool, stride 2
	
conv2	50 × 7	$\left[\begin{array}{l} 1\times 1,64\\ 3\times 3,64\\ 1\times 1,256 \end{array}\right]\times 3$

conv3	25 × 4	$\left[\begin{array}{l} 1\times 1,128\\ 3\times 3,128\\ 1\times 1,512 \end{array}\right]\times 4$

conv4	13 × 2	$\left[\begin{array}{l} 1\times 1,256\\ 3\times 3,256\\ 1\times 1,1024 \end{array}\right]\times 6$

conv5	7 × 1	$\left[\begin{array}{l} 1\times 1,512\\ 3\times 3,512\\ 1\times 1,2048 \end{array}\right]\times 3$

	1 × 1	global average pool 2-d fc, softmax

**TABLE III:** DenseNet-201

stage	output	kernels

conv1	102 × 15	7 × 7, 64, stride 2

		3 × 3 max pool, stride 2
	
dense1	51 × 8	$\left[\begin{array}{l} 1\times 1\\ 3\times 3 \end{array}\right]\times 6$

tr1	25 × 4	1 × 1, 256 and 2 × 2 avgpool, stride 2

dense2	25 × 4	$\left[\begin{array}{l} 1\times 1\\ 3\times 3 \end{array}\right]\times 12$

tr2	12 × 2	1 × 1, 512 and 2 × 2 avgpool, stride 2

dense3	12 × 2	$\left[\begin{array}{l} 1\times 1\\ 3\times 3 \end{array}\right]\times 48$

tr3	6 × 1	1 × 1, 1024 and 2 × 2 avgpool, stride 2

dense4	6 × 1	$\left[\begin{array}{l} 1\times 1\\ 3\times 3 \end{array}\right]\times 32$

	1 × 1	global average pool 2-d fc, softmax

**TABLE IV:** Pulse Frequency Mismatch Section IV-A

Experiment Type	Accuracy (ResNet)	Accuracy (DenseNet)	AUC (ResNet)	AUC(DenseNet)
Train-Time Calibration (50%)	93.31±0.65	90.83±2.68	0.987±0.004	0.982±0.005
Train-Time Calibration (100%)	**96.77±1.04**	**95.45±1.34**	**0.996±0.001**	**0.994±0.001**
Test-Time Calibration	93.25±2.91	93.53±1.52	0.989±0.003	0.989±0.002
TL with 2 Frames	94.69±2.63	94.38±2.38	0.986±0.014	0.984±0.013
TL with 10 Frames	97.11±1.11	95.64±1.42	0.995±0.003	0.989±0.006
TL with 20 Frames	97.41±0.33	95.83±0.49	0.996±0.001	0.992±0.003
No Calibration	52.35±0.88	52.33±1.10	0.927±0.001	0.938±0.010
Benchmark	99.65±0.51	99.46±0.58	0.999±3.4e-5	0.999±4.3e-5

**TABLE V:** Focal Mismatch Section IV-B

Experiment Type	Accuracy (ResNet)	Accuracy (DenseNet)	AUC (ResNet)	AUC(DenseNet)
Train-Time Calibration (50%)	94.95±0.96	95.63±0.90	0.991±0.002	0.994±0.001
Train-Time Calibration (100%)	94.37±1.53	94.68±1.73	**0.997±0.001**	**0.996±0.001**
Test-Time Calibration	**96.67±0.46**	**96.34±0.77**	0.996±0.001	0.995±0.001
TL with 2 Frames	93.51±3.03	94.07±3.65	0.987±0.011	0.980±0.021
TL with 10 Frames	96.66±0.86	95.41±1.15	0.993±0.003	0.988±0.007
TL with 20 Frames	98.22±0.35	96.62±0.53	0.997±0.001	0.993±0.003
No Calibration	83.44±1.52	85.52±0.73	0.929±0.012	0.939±0.009
Benchmark	99.65±0.51	99.46±0.58	0.999±3.4e-5	0.999±4.3e-5

**TABLE VI:** Output Power Mismatch Section IV-C

Experiment Type	Accuracy (ResNet)	Accuracy (DenseNet)	AUC (ResNet)	AUC(DenseNet)
Train-Time Calibration (50%)	98.24±0.59	**98.39±0.61**	0.999±4.1e-4	0.999±4.2e-4
Train-Time Calibration (100%)	97.06±0.54	98.15±0.74	0.998±0.002	0.999±0.001
Test-Time Calibration	**98.99±0.35**	98.26±0.21	**0.999±3.2e-4**	**0.999±3.3e-4**
TL with 2 Frames	96.85±1.05	97.09±2.05	0.995±0.007	0.997±0.002
TL with 10 Frames	98.65±0.45	98.73±0.42	0.999±7.3e-4	0.999±4.0e-4
TL with 20 Frames	99.26±0.34	98.73±0.38	0.999±5.1e-4	0.999±8.0e-4
No Calibration	86.98±1.45	84.41±1.15	0.957±0.010	0.923±0.002
Benchmark	99.65±0.51	99.46±0.58	0.999±3.4e-5	0.999±4.3e-5

## Highlights

- The most significant element of novelty: The proposed method utilizes a calibration phantom and setting transfer functions to calibrate acquisition-related data mismatches for deep learning-based quantitative ultrasound.
- An executive summary on the main results: The results showed that the proposed method can effectively calibrate pulse frequency, focal region and output power related data mismatches between training and testing data distributions.
- Highlight the implications and importance of the reported findings to the research field: The paper developed an economical technique for generalizing deep learning models for tasks in which the ultrasound scanner settings are not fixed by the operator.
